# Passing Events and Topics

**Published:** 1921-02-19

**Authors:** 


### Passing Events and Topics.

### College of Ambulance.

H.R.H. Princess Christian has accepted the position of President of the College, and has promised to attend the meetings and give such advice as she is able to.

### Borough Councils and Free Milk.

Since the Ministry of Health issued the Order which empowered local authorities to supply milk to mothers and young children in connection with maternity and child-welfare schemes, there have been different interpretations as to the class of people for whose benefit the Order was intended. The practice varies considerably in London, and now it is proposed by the Islington Borough Council to call a conference to consider the procedure to be followed in dealing with applications for the supply of milk with a view to secure uniformity of action. The Health Ministry is to be invited to send a representative. Islington Borough Council is now spending £2,000 a month in the supply of milk.

### Lectures on Tuberculosis.

Commencing on March 1, at 8 p.m., and on every subsequent Friday and Tuesday till May 3, a series of lectures will be given at the Hospital for Consumption, Brompton, intended for trained nurses, health visitors, and social workers. The fee for the course is £1 1s., or with demonstrations and examinations £2 2s. For single lectures the fee is 2s.

### The Royal Chest and Great Northern Amalgamation.

As we go to press a meeting of Governors is being held at the Great Northern Central Hospital to consider and, if thought fit, to pass a resolution approving of the recommendation of the Committee of Management in respect of the absorption of the Royal Chest Hospital, City Road. The amalgamation has already been agreed to by the Governors of the last-named institution.

### Medical Education in China.

An important meeting of members of the medical profession is to be held on Monday, February 21, at 5.30 p.m., at the House of the Royal Society of Medicine, 1 Wimpole Street, London, W. 1, to emphasise the great progress which has been made in China within recent years in the development of medical education, in which British and American practitioners have taken a leading part. The chair will be taken by Sir Donald Macalister, K.C.B., M.D., President of the General Medical Council, and an address will be given by Dr. Harold Balme, F.R.C.S., Dean of the Faculty of Medicine of the Shantung Christian University, Tsinan. Amongst others who hope to be present are the Rt. Hon. Sir Clifford Allbutt, K.C.B., M.D., Sir Alfred Pearce Gould, K.C.V.O., M.S., Sir Francis Champneys, Bart., M.D., and the Rt. Hon. Sir John Jordan, G.C.I.E., K.C.M.G. The newly appointed Chinese Minister will be represented by Mr. Owyang King, First Secretary of the Legation.

